# How do decision-makers use evidence in community health policy and financing decisions? A qualitative study and conceptual framework in four African countries

**DOI:** 10.1093/heapol/czaa027

**Published:** 2020-06-09

**Authors:** Meghan Bruce Kumar, Miriam Taegtmeyer, Jason Madan, Sozinho Ndima, Kingsley Chikaphupha, Aschenaki Kea, Edwine Barasa

**Affiliations:** c1 Community Health Systems Group, Department of International Public Health, Liverpool School of Tropical Medicine, Liverpool, UK; c2 MARCH Centre, London School of Hygiene and Tropical Medicine, London, UK; c3 Tropical Infectious Disease Unit, Royal Liverpool University Hospital, Liverpool, UK; c4 Centre for Health Economics at Warwick, Warwick Medical School, University of Warwick, Coventry, UK; c5 Community Health Department, Faculty of Medicine, University Eduardo Mondlane, Maputo, Mozambique; c6 Research for Equity and Community Health (REACH) Trust, Lilongwe, Malawi; c7 School of Public and Environmental Health, Hawassa University, Hawassa, Ethiopia; c8 Center for International Health, University of Bergen, Bergen, Norway; c9 Health Economics Research Unit, KEMRI-Wellcome Trust Research Programme, Nairobi, Kenya; c10 Centre for Tropical Medicine, Nuffield Department of Clinical Medicine, University of Oxford, Oxford, UK

**Keywords:** Economic evaluation, community health, evidence-based policy, health financing, Ethiopia, Kenya, Malawi, Mozambique

## Abstract

Various investments could help countries deliver on the universal health coverage (UHC) goals set by the global community; community health is a pillar of many national strategies towards UHC. Yet despite resource mobilization towards this end, little is known about the potential costs and value of these investments, as well as how evidence on the same would be used in related decisions. This qualitative study was conducted to understand the use of evidence in policy and financing decisions for large-scale community health programmes in low- and middle-income countries. Through key informant interviews with 43 respondents in countries with community health embedded in national UHC strategies (Ethiopia, Kenya, Malawi, Mozambique) and at global institutions, we investigated evidence use in community health financing and policy decision-making, as well as evidentiary needs related to community health data for decision-making. We found that evidence use is limited at all levels, in part due to a perceived lack of high-quality, relevant evidence. This perception stems from two main areas: first, desire for local evidence that reflects the context, and second, much existing economic evidence does not deal with what decision-makers value when it comes to community health systems—i.e. coverage and (to a lesser extent) quality. Beyond the evidence gap, there is limited capacity to assess and use the evidence. Elected officials also face political challenges to disinvestment as well as structural obstacles to evidence use, including the outsized influence of donor priorities. Evaluation data must to speak to decision-maker interests and constraints more directly, alongside financiers of community health providing explicit guidance and support on the role of evidence use in decision-making, empowering national decision-makers. Improved data quality, increased relevance of evidence and capacity for evidence use can drive improved efficiency of financing and evidence-based policymaking.

Key Messages

- The use of evidence in national community health policy and financing decisions is limited by its perceived poor quality and the capacity of decision-makers to use it.
- Most existing evidence is perceived as of limited relevance to domestic decision-making; it is used more by global financiers of community health.
- Decision-makers emphasize increasing coverage of or access to services community health services—quality is rarely mentioned as a funding priority.
- Stopping an established approach to community health (disinvesting) in favour of another more economically viable approach is seen as politically challenging even when evidence exists.

## Introduction

Well-resourced close-to-community (CTC) health workers can deliver high-quality care; there is extensive, robust, project- and trial-based evidence for this across a range of settings and disease areas, as shown in a set of recent reviews of community health programmes (Chou *et al.*, 2017; Freeman *et al.*, 2017; Jennings *et al.*, 2017; Perry *et al.*, 2017; Sacks *et al.*, 2017; Schleiff *et al.*, 2017; Scott *et al.*, 2018). Armed with this evidence, extending access to primary health care through CTC cadres with an aim of universal health coverage (UHC) has long been used as an approach and lately becomes a priority in many countries (Wang *et al.*, 2016; Bhutta, 2017; Javanparast *et al.*, 2018). The World Health Organization has supported operationalization of extending access in this way through the development of guidelines for national CTC programmes (Cometto *et al.*, 2018). Yet in many health systems, community health remains perceived as an extension of the ‘formal’ system rather than a core, integrated service delivery platform (Theobald *et al.*, 2015; Schneider and Lehmann, 2016; Tseng *et al.*, 2019).

Economic evidence should play a predominant role in the integration of community health into wider health systems, due to the need for trade-offs between different health investments and competition for limited resources. There is a small but growing body of economic evidence on community health programmes, recently presented in a systematic review by Nkonki *et al.*; like the evidence on quality described above, the authors state that most evidence is ‘from small scale and vertical programmes’ (Nkonki, Tugendhaft and Hofman, 2017). Once community health programmes start operating at scale, quality of care and performance of CTC providers do not always live up to their potential (Kok *et al.*, 2015; Silva *et al.*, 2016; Yourkavitch *et al.*, 2016; Ballard *et al.*, 2017; Phiri *et al.*, 2017; Lehmann *et al.*, 2019). The limited economic evaluations of the quality of large-scale CTC programmes generate uncertainty about the value of this investment; studies on the costs of large-scale CTC programmes (Vaughan *et al.*, 2015; Barger *et al.*, 2017; Daviaud *et al.*, 2017; Nkonki *et al.*, 2017; Taylor *et al.*, 2017) have rarely incorporated data on the quality of care. In assessing outcomes, incorporating quality into economic evaluations of CTC programmes is challenging because of difficulty in defining the quality of care for CTC interventions and the complex causal pathways between CTC quality of care measures and health outcomes. Poor data quality affects measurement across both costs and outcomes (Yourkavitch *et al.*, 2016; Regeru *et al.*, 2020).

As an extension or even marginalized aspect of the healthcare system, community health decision-making does not benefit from the many formal procedures for generating and using evidence that have been developed in the wider health sector. For example, there is a gap in community-focused financing literature; Scott *et al.* showed that, out of 122 publications on the ASHA programme in India between 2005 and 2016, only five dealt with financing (Scott *et al.*, 2019). Where it exists, community financing literature often focuses more on community-based micro insurance schemes rather than macro financing of community health programmes, despite a predominance of external financing in this space (McCollum *et al.*, 2018c; Agarwal *et al.*, 2019). Because potential users of evidence often perceive community-level care as ‘free’ to the system, this limits the commissioning of economic evidence at local and national levels. Similarly, in the wider sector, the broad literature on proceduralism focuses on formalized processes for evidence use, consultation and transparency (Barasa *et al.*, 2015)—yet in community health as a sub-sector, these processes are not well established. As such, even when evidence about community health interventions is available, this evidence may be underutilized in decision-making. In the absence of sufficient procedures (the largely external), investment in community health seems to be driven by ideology and global movements over evidence. A closer look specifically at community health decisions within the health sector is imperative given the relative marginalization of community health as an extension of the health system and its reliance on external financing in many low- and middle-income countries (LMICs) (Theobald *et al.*, 2015; Javanparast *et al.*, 2018; Tseng *et al.*, 2019).

This multi-country qualitative study was designed to understand the role of evidence in how decisions are made for community health financing and policy at national and global levels. We focus our study in four countries (Ethiopia, Kenya, Malawi, Mozambique) that have large-scale public sector community health programmes that remain highly dependent on external financing. In key informant interviews with national and global funders, policymakers and researchers, we set out to understand what evidence is used and by whom, and when and what additional evidence could improve the efficiency of community health decision-making in settings where CTC providers are an integral part of the health system.

## Methods

We used a qualitative cross-sectional approach to understand the use of economic evaluation evidence in community health priority setting and financing. Qualitative methods were utilized to allow for an understanding of the process (how things are currently working), stakeholders (who is involved) and wider decision space (role of the health system and context). Given the limited number of individuals involved in policy and financing decisions and their seniority, key informant interviews were selected as the most appropriate methodology to extract relevant information. Data were collected between November 2017 and November 2018.

### Study sites and sampling

Forty three key informant interviews were conducted with purposively sampled decision-makers involved in community health policy and financing decisions at national and global levels based on the sampling frame shown in Table 1. We selected countries with national community health programmes in Africa that were part of the REACHOUT consortium: Kenya, Ethiopia, Malawi and Mozambique^1^ (REACHOUT, 2013). Respondents included national and sub-national Ministry of Health staff involved with community health financing and/or programming and implementers of large-scale community health programmes. The global interviews included institutional financiers of community health, community health researchers and normative agencies. These respondents were selected to represent those making community health policy and financing decisions in the selected REACHOUT countries, generating evidence to inform the decisions, and those affected by the decisions through involvement in translating policy to practice.

**Table 1:** Sampling framework

Category	Possible respondents	Location	Eligibility criteria
Global funders and policymakers of community health	USAID, Global Fund, UNICEF, WHO, UN Special Envoy, Financing Alliance, DfID, Health Systems Global CHW TWG members	Global—mix of remote and in-person	Funders of cases identified in the literature review
National/sub-national CTC programme decision-makers	National Community Health Unit, National Division of Standards, District/County Community or Quality Focal Point, NGOs (as appropriate)	2–4 per country at relevant levels In-person	Identified by REACHOUT country teams Involved in: policy, financing, and/or programming decisions for CTC providers
Community health researchers and implementers	REACHOUT country Principal Investigator;Local academic or NGO-based researchers (international only if embedded)	2–3 per country In-person	Working on REACHOUT project for 3 or more years OR Researching CTC providers for 3 or more years

### Data collection, management and analysis

Interviews were guided by a semi-structured topic guide, which was piloted in Kenya before use (available in Supplementary File S2). We asked respondents’ questions about their community health experience, about domestic and external financing for community health in their setting, and, using quality improvement as a case example of a project, about evidence needs, evidence use, and financing mechanisms related to decision-making and the (community health) decision space.

In all cases except two in Ethiopia and two in Mozambique, interviews were conducted in English by the corresponding author. In those four interviews, local researchers with prior experience in qualitative methods and community health were trained in the interview content and objectives and conducted the interviews.

Thirty nine of 43 total interviews were digitally recorded and transcribed by local researchers in each country (where they were conducted in another language, they were transcribed in the local language and then translated); the remaining respondents asked not to be recorded and interviewer notes were included in lieu of a transcript. Code frame development was done deductively according to the objectives of the study: understanding decision-maker perspectives on quality and understanding the use of evidence in decision-making for community health financing and programming. Additional codes (particularly, detailed information around financing and economic evidence) were added inductively in the course of the analysis as they had arisen due to the open questioning style used in the interviews (Gale *et al.*, 2013) (for full code frame, see Supplementary File S3). Analysis was assisted by NVivo11 software, and for each theme, relevant quotes were examined to generate a draft narrative. A thematic framework approach was used for the analysis (Gale *et al.*, 2013). Given that there was a single lead researcher conducting interviews and coding, quality assurance was done in the following ways: (1) review of selected transcripts by senior authors; (2) coding workshop with colleagues where multiple individuals coded transcripts to ensure inter-coder reliability; and (3) discussions with and feedback from research partners/co-authors in each country on emerging themes. For non-recorded interviews, the notes were included as transcripts and coded in the same way as verbatim transcriptions described above; direct quotes from these interviews were not included due to the risk of misrepresentation of exact wording.

## Results

### Respondent characteristics

A total of 43 key informant interviews were conducted with purposively sampled respondents working in community health at national and global levels. In total, these were: global (*N* = 11), Ethiopia (*N* = 10), Kenya (*N* = 7), Malawi (*N* = 6) and Mozambique (*N* = 6); descriptions of respondents are shown in Table 2. There was a focus on policy and financing decision-makers, with the latter being over-represented at global level due to the predominance of external financing in this area. Implementers and health workers represent the individuals who translate decisions into practice/action and have a perception of how and if their evidence gets used in this process. Of the researchers, who represented a smaller proportion of the total sample, two were economists and the majority was working more broadly on implementation research, governance, feasibility and process evaluations in the CTC space.

**Table 2:** Respondent characteristics^a^

Level/country	Programme policy	Programme financing	Researcher	Implementer/health worker	Total
Global	1	5	2	3	11
National/Ethiopia	3	1	1	5	10
National/Kenya	3	1	2	3	8
National/Malawi	2	2	1	3	7
National/Mozambique	2	1	1	4	7
Total	43				

a Respondents were allowed in exceptional cases to be included in more than one category or quota.

Institutions represented at the global level included: UNICEF, World Health Organization; Global Fund to Fight AIDS, Tuberculosis and Malaria; Global Financing Facility for Women, Children and Adolescents; Last Mile Health; Financing Alliance for Health; United Nations’ Special Envoy for Health; Community Health Impact Coalition; United States Agency for International Development; South Africa Medical Research Council; and John Snow International representing Maternal and Child Health Integrated Program; out of this group, implementers are those organizations that deliver community health programmes in country. Institutions represented at country level are national and sub-national government staff as well as non-governmental organizations (NGOs) and International Organisations as relevant to the community health planning, financing and delivery in each context.

### Evidence use in national-level decisions for publicly funded programmes

The reported use of economic evidence in health policy and financing decisions varied by country but was generally informal and motivated by individuals instead of systems. Ethiopia demonstrated the most formalized processes and procedures for the use of economic evaluation in the health sector at the national level, with a separate department inside the Federal Ministry of Health’s Planning Directorate responsible for using and assessing economic evidence (particularly finance data from National Health Accounts and evaluation data from Public Health Research Institute). No study countries systematically required the use of economic evidence in decision-making for as a formal stage in public policy or financing decisions for community health. Community health systems were, in the views of most respondents, an extension of the health system rather than a core part, evidenced in part by the title of CTC workers as ‘extension workers’ in some settings. As such, community health was viewed as a lower priority than other health areas in terms of commissioning evidence, and related decision-making was less restricted by formalized processes and requirements for evidence. In the absence of these governing structures, change was often described in our data as driven by individual leaders and/or the desire for political advantage instead of evidence, as in this case from Kenya:

*I haven’t seen anyone talking about an incremental approach [to policy change in community health]; I have just seen the type like Kitui [County] where you [leaders] decide today: ‘I’m going to do this and I’m going to put this money’* (community health researcher, Kenya).

The most commonly available evidence of impact or benefits of community healthcare investments at national level, understood as programme performance by the majority of respondents, was generated by CTC health workers through routine monitoring and evaluation. However, these routine data were not thought by most to be reliable enough to support decisions; improving the quality of routine CTC data was considered by several respondents to be a prerequisite to its use. This was compounded by the fact that these data are often paper based (community data are reported in District Health Information Software 2 only in Kenya and Ethiopia, and even these are often incomplete), so the process of obtaining performance data from this source may have prohibitive time cost. A sub-national key informant in Mozambique told us of frustrations in trying to get and use routine community health programme data in their work:

*The APE [or CHW] is producing data in a useful way but this information I feel that, I do not know where it is going because I do not have a report of what happens to ‘my’ information*. *I get a bit confused because there is no transparency of where [that] information goes*. *When I consult the Ministry, they say that it is used by the programmes, but we at the level of the province we do not feel that* (policymaker, Mozambique).

Few national-level respondents talked about using cost-effectiveness evidence to inform decisions, though in Ethiopia there were several who mentioned aspirations to generate their own cost-effectiveness data for projects and new programmes. The limited number who mentioned them stated that cost-effectiveness studies, where available, are not seen by national policymakers as addressing budget constraints, as they do not address real constraints on available financing. This was summarized by a respondent in the Federal Ministry of Health in Ethiopia as follows:

*The results they submitted to us [show] if the implementing second generation is the extension program cost effective? But it needs really further discussion and also policy dialogue also with some stakeholders … it’s more expensive … I think we need more data like for example if we implement second generation extension program all over the country how much cost it will take and the other thing what are the health gains in this amount of investment.*

In contrast, several respondents discussed costing data being used alone without effectiveness data. These data were used mainly to fundraise, through approaches like gap analyses, and to decide whether to expand coverage of the CTC programme.^2^ Despite expanding coverage or ‘extension’ of services being a stated aim of community health programmes in all study countries, no respondents directly stated a need for evidence on the equity of community health services. Respondents used ‘coverage’ to address primarily geographical equity considerations, but no direct mention was made of other aspects of equity. Healthcare workers in Ethiopia described the equity-linked challenges in their community work:

*To work on quality, the problem we face is that patients are found in geographically difficult areas … so that makes problems to communicate with us*.

Among policy makers, there were several mentions of the challenge of allocating a limited budget across many interventions. Trying to achieve allocative efficiency is a potential entry for effectiveness evidence to identify the best investments. However, instead of providing incentives to focus on priority setting, allocation of resources was linked to coordination between funders and partners to cover the different aspects of comprehensive but unfunded annual plans. In this way, coverage sometimes meant avoiding the duplication of efforts in investments rather than increasing access to healthcare services. An implementer in Mozambique stated the challenges of prioritizing investment in community health in their planning processes simply:

*[Access is prioritised over quality] – and this is linked to resources; if resources are slim and you have to go strengthen at the community level or the health facility level, what do you do?*

### Evidence use in funding applications

The influence of external financing and donor priorities on community health decisions came out strongly in the data. In the study countries, external financing is a majority of the community health financing, yet it was seen as unpredictable and (often) having limited flexibility. A financing agency key informant in Kenya described the role of external financing on community health:

*… the disadvantage of being off budget is you are working outside the system. Yeah, it’s a parallel system which is unhelpful in many ways and complicates things. That’s one of the causes why community health care is funding ‘off budget’ mainly and by donor funding*.

Each donor and their priorities were described as changeable and contingent on other fiscal planning and calendars—yet they put pressure on national government to adapt to and often adopt their priorities. For many national level key informants, the predominance of external financing brought about a lack of motivation and/or space to drive the agenda in their own health sector.

*You see like right now say USAID has money and all their money goes to partners … the partners need to implement what USAID and government have agreed on; so theoretically that is what happens but we know mostly it is pushed by USAID and we follow that and because the counties just want the money, they will say: ‘it’s fine let’s go ahead’ …* (community health researcher, Kenya).

External financing was seen to limit the value of economic evidence to government staff; governments are desensitized to the full costs of these programmes and in some cases view the international priorities as ‘pre-vetted’ for impact. In addition, these programmes are often tightly earmarked and thus evidence becomes irrelevant until the project funding period is over. Externally funded NGO-led projects are often required to report programmatic costs, but governments are not directly trading off these investments against other possible programmes and the focus on sustainability is limited. Instead, the Ministries of Health are occupied with the coordination of programmes contingent on external funding cycles rather than driving implementation based on (local) evidence, as described in Mozambique:

*I see that the Ministry of Health goes with this programme but at the same time they are not preparing themselves for taking over. They still rely on the partners; that is the big issue. This programme depends too much on the partners* (community health implementer, Mozambique).

### Evidence use in priority setting for global financing and the role of global agendas on domestic financing

Globally, there is a stated or ‘on paper’ agreement about the need for evidence to underpin decisions, in part to address fairness concerns among those competing for financing. These fairness concerns were restated in calls by national-level respondents for transparency in financing decisions by global-level financing mechanisms. Despite this stated commitment, political processes and prioritization exercises precede the evidence-based decisions in several cases. For example, the initial allocation of funds to human immunodeficiency virus/acquired immune deficiency syndrome, tuberculosis and malaria for each country from the Global Fund is made according to a formula. Subsequently, community health, as a component of the health systems strengthening envelope within the country allocation, has to ‘fight’ for resources from these disease areas. Similarly, in the Global Financing Facility of the World Bank, the reasons for selection of the priority countries were opaque, according to this key informant:

*How the 16 countries were selected, I’m not completely sure … well, partly it was our priority countries because there was a political economy angle to the countries from the donor side, so there’s also these countries themselves who say they want … to be part of it so it will require they speak for themselves* (key informant, global).

The biggest global items influencing community health, UHC and the United Nations’ Sustainable Development Goals for health were mentioned in each of the study countries by at least one respondent despite there being no direct question about it. Of the respondents who mentioned it, all national policy makers of funders of community health, several did not have a clear definition of UHC, potentially limiting its efficacy at motivating financing or policy shifts. However, they stated that pressure from global stakeholders towards UHC is increasing, without clarity what evidence would be needed to measure progress towards this global goal. The perceived relationship of UHC to economic evidence was limited and primarily related to access to financial protection for community members, as stated by a policymaker in Kenya:

*… the Permanent Secretary and the Cabinet Secretary they were really looking at how community strategy can be used to reach people in the coverage of the National Hospital Insurance Fund.*

The evidence being generated to support these global agendas was perceived by the majority of respondents to focus predominantly on feasibility and impact evaluations of small-scale pilots and programmes in specific locations, sometimes called ‘pilot-itis’. This led respondents to be concerned that the resulting evidence is not relevant to other contexts, even within the same country. In those sites where CTC providers have greater curative responsibilities, particularly Ethiopia, respondents felt that a lot of community health evidence was not relevant to their ‘highly skilled’ CTC providers, so they tended to call for more ‘local evidence’. Seemingly in contrast, in Kenya, national policymakers felt that devolution of decision-making to sub-national administrative units at county level might have led to the fragmentation of evidence needs, with demand for research and evaluation from each county.

### Quality of care not a priority in the assessment of investments in community health

Quality of CTC care was usually termed ‘performance’ by respondents, and most respondents had low expectations of quality and performance. By the majority of respondents, CTC care was viewed as a means of expanding ‘coverage’, focusing largely on geographic barriers to care (e.g. >5 km to a health facility) rather than social, economic or other barriers to equitable health care. They viewed this as reasonable given the relatively simple tasks allocated to most CTC providers and their limited levels of education and formal health training. Community health financing decisions, both domestic and external, have similarly emphasized the requirement for geographic spread over quality, and this was also a focus of responses that equated coverage with quality, with no mention of ‘effective coverage’:

*We’ve seen that they [the donor] are very much like we want a number of children immunised to be such and such; it’s not about quality its really about numbers and coverage* (community health implementer, Ethiopia).

At the national level, decision-makers stated that the aspects of quality they would like to have evidence of included: improving health outcomes (in all countries), data quality (mainly Malawi and Kenya, with two mentions in Mozambique), ownership by and accountability of services to citizens (in all study sites except Ethiopia). Most stated that quality could be improved through better supervision and policy changes. In Ethiopia, respondents were more likely to mention health benefits in specific health areas and in some cases to describe meeting system-wide targets as a proxy for quality (e.g. quotas for percentages of deliveries attended by a skilled birth attendant). Across countries, evidence for improved quality that would be acceptable to participants included: changes in reporting rates for routine data on community health services, increased demand for services at primary healthcare facilities, decreasing burden of disease and CHW/community satisfaction. However, many national-level key informants acknowledged that quality was difficult and expensive to measure, as the challenges with routine data meant that understanding the quality of care was perceived to require additional, non-routine data collection. As such, most respondents also had limited expectations for evaluations to be able to incorporate robust evidence on quality.

The design and integration of quality management structures in the Ministries of Health appeared to influence the appetite for economic evidence examining quality or performance. In Ethiopia and Mozambique, quality was a small part of the job description of technical staff in well-funded disease departments (e.g. malaria). In contrast, in both Kenya and Malawi, healthcare quality and standards were managed by a stand-alone department, supporting dedicated staff who promoted the quality agenda in evidence and decisions across the sector. Yet in these countries, quality management staff were sometimes marginalized or excluded from decision-making due to a lack to technical health area focus, as shown in this example from Malawi:

*That was our original plan to have quality improvement persons in each [technical] department; we have one meeting and then the directorate [of quality management] calling them but of course nobody showed up and that is the challenge these departments always have* (programme implementer, Malawi).

Yet even where there is an independent quality structure, getting that structure to consider the ‘extension’ of their mandate to community level could still prove a challenge, as continuing with the Malawi example illustrates:

*They [the directorate of quality management] … initially they were saying—‘why should we talk about the community?’ and I said ‘no, then you are joking*’ (policymaker, Malawi).

The same was true in Kenya, where the national Kenya Quality Model for Health had not been functionally extended to the community level or even disseminated by the National Department of Quality and Standards.

### Non-evidentiary influences on decisions

At the immediate decision level, almost every discussion came back to a combination of limited relevant evidence and limited capacity to use the evidence that exists. This limited capacity was described as leading to a lack of demand for evidence and limited resources dedicated to commissioning or generating evidence, creating a vicious cycle. It also creates a vacuum that advocates of particular approaches or programmes were described as filling with their own priorities, through power and their political savvy. Decision-makers try to juggle this influence alongside many other non-evidentiary limitations:

*… the decision makers, are they able to use comparative cost analyses against different programme and make sort of an effectiveness decision, sort of that? And I think the answer is no, that they will only use the data for decision making not in a vacuum, there’s like a million other constraints ….* (community health implementer, global).

At the national level, the role of power over evidence appeared to be related to the degree of decentralization of the health sector, but this relationship was complex; decentralization was described as allowing space for more levels of ‘politics and power’, while also potentially increasing accountability due to proximity between voters and decision-makers, so it did not play out the same way in different locations but was dependent on individuals. Across the countries, contextual factors including varied responsibilities of community health workers, limited formal evidence consideration in most annual work planning procedures and complex interactions between Ministries of Health and of Finance were seen to influence the likelihood of evidence use in decisions. Similarly, a couple global respondents identified that where programmes were not nationally led (but rather NGO led), the geographic impacts would be piecemeal and may not be generalizable across the country.

Finally, interactions (i.e. power) and political viability were key to understanding decisions—both among global funders ‘competing’ for implementation space in priority countries and among national policymakers looking for re-election for themselves or their party, as well as between these global- and national-level actors. This links to the negative public opinion that faces national and sub-national decision-makers who try to use evidence to justify removing established services, or to disinvesting, as this Ethiopian policymaker described:

*Actually, it is very difficult for communities, for example some strategies being implemented for the last ten or fifteen years, the community is highlight dependent on that so there may be a resistance with the community [to stop funding something].*

Despite this, global (international and bilateral) influence on national priorities was consistently present in the data and continues in large part because it comes with financial support—and expectations of delivering on donor priorities.

## Discussion

This multi-country analysis on the use of evidence in community health in LMICs highlights a gap around the use of economic evidence in financing and policy decisions. We find limited use of evidence in decision-making for community health and confirm findings from other studies that power and politics have noteworthy influence on priority setting. In explaining why evidence is not used, respondents described a lack of ‘useful evidence’, with available evidence perceived as not generalizable and not responding to the resource limitations on the ground, as well as limitations in capacity to evaluate and apply the evidence meaningfully. Due to a predominance of external financing of CTC programmes, national decision-makers are desensitized to the full costs of programmes. Donor priorities often fill the vacuum created by ‘useful evidence’ gaps, and this is reinforced by the unpopularity of disinvestment among constituents. CTC providers continue to be viewed as a means of increasing access to primary healthcare services; increased coverage of health services is the main benefits that decision-makers expect from investment in community health, with quality (or effective coverage) and equity largely absent from participant-identified evidence gaps.

**Figure 1:**
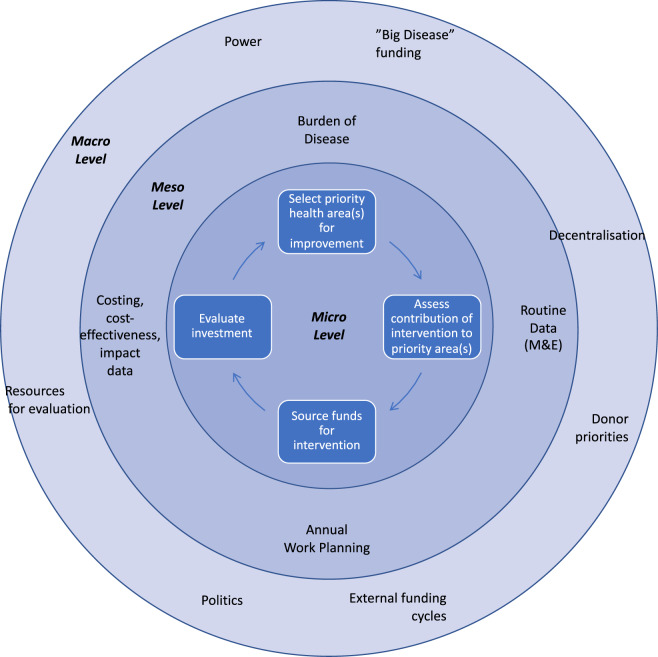
Conceptual framework for influences on community health programming decisions.

Evidence use in community health programming is constrained and influenced by contextual factors unrelated to the relevance and quality of the evidence. We conceptualize the influences on such decisions as coming from three levels: micro, meso, and macro as derived from the results as shown in Figure 1 (Caldwell and Mays, 2012). In the inner circle or micro level, we show the ‘ideal’ of evidenced-based policy setting and implementation, including priority setting, evidence assessment, decision-making and financing.

At the meso level, we show the constraints on the ideal micro or decision level. The first constraint is environmental/epidemiological and service data availability and quality. At the meso level, routine community data quality is poor and most countries do not have recent sub-national data on epidemiology and costs of interventions. The second constraint stems from a lack of processes and procedures (e.g. where annual work planning is done primarily related to historical expenditure and programming). Marginalization of community health from the ‘formal’ health system means fewer formalized procedural requirements for evidence use in decision-making and less commissioning of such evidence (in comparison with other health areas). Because of these limitations in community health in many countries, even where evidence exists, it is perceived as irrelevant and decision-makers are not encouraged to use it. The third limitation is capacity for evidence selection, understanding and use in community health decision-makers; this is a finding from consistent with wider studies in LMIC health systems (Stansfield *et al.*, 2006; Wickremasinghe *et al.*, 2016; McCollum *et al.*, 2018b,c; Vanyoro *et al.*, 2019). Comprehensive planning for community health programmes would involve decision-makers assessing an extensive set of routine data from health information systems that include: census, vital events, monitoring, public health surveillance, resource tracking, facility-based service statistics and household surveys (Stansfield *et al.*, 2006). Analysing these data, setting priorities and then aligning priorities to available resources are essential skills (Schneider and Nxumalo, 2017), and indeed in a recent study in Zambia, managers indicated that costing information highlighted priorities for more efficient use of resources in immunization programming (Feldhaus *et al.*, 2019). However, capacity strengthening around these transferable skills is rarely funded by vertical programmes, the main source of external financing for community health programmes (Conn *et al.*, 1996). Increased capacity could increase the appetite for evidence and could be reinforced by involving policymakers in research activities whenever possible and bringing them to the ground to see what ‘impact’ and ‘benefits’ means to workflows in the health system and livelihoods in the wider communities, potentially overcoming political barriers to evidence use, similar to what Schneider proposes related to community health governance (Schneider, 2010).

Finally, at the macro level or outer circle, decisions are influenced by health sector structures, decision and fiscal spaces, funders and their priorities (WHO, 2014; Katahoire *et al.*, 2015a,b; Greenhalgh *et al.*, 2017; Pfadenhauer *et al.*, 2017; Rajkotia, 2018). At the macro level, global institutions that finance community health programmes are more likely to formalize the use of economic evidence. However, as a result of the levels of external financing, priorities of global institutions then have an outsize influence on domestic agendas, delinked from local evidence and need in many cases.

Overall, this builds on the work of McCollum *et al.* from the Kenyan context showing that a lack of high-quality, relevant evidence and limited capacity to use it, compounded by external influences, allows power and politics to trump evidence use in many community health programming decisions (McCollum *et al.*, 2018c). We add the generalizability of these findings beyond priority setting and into non-devolved systems. In this conceptual framework, the different aspects highlighted at each level illustrate where and how evidence could be leveraged, if available, to overcome the role of power and politics in decision-making to improve the targeting of services and efficiency of the investments in health.

A core tenet of economics is that a decision-maker ought to take into account both the benefits of the intervention and the resources required to achieve those benefits and then to compare these relative to other potential investments and make a rational choice (Varian, 1992). Our findings that respondents do not perceive current cost-effectiveness studies to reflect their budget constraints suggest that, at a minimum, available studies do not accurately reflect the opportunity costs, perhaps due to inappropriate thresholds. Indeed, much critique of various thresholds (and in some cases, any thresholds at all) for cost-effectiveness has been levelled in the literature over the last 10 years (Newall *et al.*, 2014; Marseille *et al.*, 2015; Ochalek *et al.*, 2015, 2018; Woods *et al.*, 2016). In response to the push for UHC, the last 5 years have seen the development of a dizzying suite of investment cases, strategies targeting non-traditional donors and innovative approaches to promote consistent, sufficient financing of community health (Singh *et al.*, 2013; Global Financing Facility, 2016; Community Health Financing Compendium, 2017; Fernandes and Sridhar, 2017; Chou *et al.*, 2018; E&K Consulting, 2018; Community Health Roadmap, 2019). In most cases, this represents progress towards integration of community health into broader health systems, though priorities often continue to reflect donor concerns (likely in response to the fact that community health systems are still primarily funded by external financing in most countries). However, it is not clear who is the decision-maker that is intended to be influenced by many of these cases and studies. Many of them target that the Ministries of Finance and CTC programme leaders are rarely explicitly considered, nor are sub-national decision-makers, despite an increasing emphasis on decentralizing decisions in LMIC health systems (Bossert and Mitchell, 2011; Otiso *et al.*, 2017; McCollum *et al.*, 2018a; Abimbola *et al.*, 2019). For this powerful evidence to be used and useful, it must consider the decision-maker more explicitly and the constraints on their decision, e.g. through budget impact analysis rather than simply reporting incremental cost-effectiveness ratios against thresholds (Revill *et al.*, 2014; Bilinski *et al.*, 2017; Robinson *et al.*, 2017; Ochalek *et al.*, 2018).

As with any multi-country study and qualitative studies more generally, there are challenges to generalizability due to the contextual variation. However, the results were generally consistent enough to suggest actions for researchers and to commissioners and users of economic research evidence in the community health space. The selection of countries from within the REACHOUT consortium near the end of that programme period might have increased some of the key informants’ consideration of and awareness of community health issues as part of the wider healthcare system in comparison to others in the region. The highly variation in degree of decentralization of community health decisions could have also created less convergence around evidence use. In terms of positionality, the collection of data by a non-local researcher might limit the willingness of some respondents (especially government staff) to be fully frank and, similarly, conducting interviews in English might have limited the nuance available to participants with more limited language proficiency.

## Conclusions

In summary, there is ample room to improve and increase evidence use in community health programming and financing decisions. The goals of the health sector are in improving population health and health outcomes; additional benefits of improved quality of CTC health worker services are intrinsically valuable but even more complex to measure—aspects such as trust, motivation, inclusion and adherence. Thus, decision-makers focus on coverage as the priority benefit that they would like to see represented in evaluations of community health programmes, yet have limited resources to commission or undertake evaluations, and limited pressure to use the results. Politics further constrains decisions primarily in two ways: first, hardware investments such as hospitals, vehicles and equipment are easy election ‘wins’, and second, removing established services that are less (cost-)effective is politically challenging, even if evidence exists. If researchers and community health decision-makers can bridge these gaps between them, the important value of evidence in improved community health programming and therefore improved population health will begin to be realized.

However, all potential approaches will have to overcome weaknesses in quality of available data, limitations in decision-maker capacity and concerns about applicability of evidence expressed by respondents in this study.

## Supplementary Material

czaa027_supplementary_data

## Footnotes

1 REACHOUT is a multi-country research consortium focused on the quality of community health that worked from 2014 to 2019 in six countries (Ethiopia, Kenya, Malawi and Mozambique in Africa and Bangladesh and Indonesia in Asia). For this piece of work, we focused on the African countries (for details on the community health programmes in the four study countries, see Supplementary File S1).

2 Expanding coverage was used to mean either adding human resources in existing sites or expanding geography of the programme.
