# Supplementary material for: How do decision-makers use evidence in community health policy and financing decisions? A qualitative study and conceptual framework in four African countries
Source: Health Policy Plan. 2020 Jun 9;35(7):799–809. doi: 10.1093/heapol/czaa027 (PMC7487332; doi:10.1093/heapol/czaa027)
Supplement: czaa027_supplementary_data [file czaa027_supplementary_data.zip › MKumar_suppl material_1_community health systems.pdf]

Study sites

As stated in the main article text, the study sites included the following countries: Ethiopia, Kenya, Malawi, and Mozambique, in addition to global-level interviews. Each of these countries was selected for two reasons:

1. Incorporation of large-scale community health programs as part of the national UHC strategy; and
2. for their representation in the REACHOUT consortium as involved in community health action research in multiple sites or locations.

All countries have community health programs that are wholly or partially government-led. However, the typology of the community health workers (responsibilities, professionalization, remuneration, etc.), structure of the programs, and level of integration with the wider health care system was variable. In this supplementary material, we give a brief description of this variation to provide greater context for the findings. In each country, ethical approval was granted and details of these approvals can be found in Supplementary File 4 to the main manuscript.

Description of the community health systems in each study site

Descriptions of community health worker typologies tend to incorporate some or all of the following dimensions: responsibilities and relationship to community and health facilities, selection and recruitment, training and supervision, remuneration and supplies. These have been described in detail by many publications, including those of the REACHOUT consortium and by the author as part of earlier work (Lewin *et al.*, 2010; Kumar *et al.*, 2014; Mireku *et al.*, 2014; Nyirenda *et al.*, 2014; Give *et al.*, 2015; Kok *et al.*, 2015; Mahmud *et al.*, 2015; Kane *et al.*, 2016; Olaniran *et al.*, 2017; Tumbelaka *et al.*, 2018; Ormel *et al.*, 2019).

Here I provide a brief description of the community health worker typologies and systems in each study site as is relevant to understanding of the findings presented in the main manuscript; further details on the typologies of the CTC providers can be found the supplementary material to a recent publication by the author (Kumar *et al.*, 2019).

Ethiopia

In Ethiopia, community health is fully institutionalized under the Ministry of Health. The main professionalized cadre of CTC providers are an all-female group called health extension workers. By policy, they are selected by their communities and two serve each community (approximately 5,000 people) at any given time (Ministry of Health, 2007). They receive training of one year in 16 "essential packages", including preventive and curative care for maternal and child health, as well as hygiene, disease prevention, and health education. They serve the community from Health Posts as well as doing household visits. They are supported by unpaid CTC providers called the Health Development Army and 1-to-5 group leaders who are the heads of model households (Datiko *et al.*, 2019). Health Extension Workers are supervised by Health Centre staff, the lowest level facility of the primary care system in Ethiopia, and *woreda* or district staff also have less frequent supervisory role. Successes of the health extension programme in various sites and health service areas are well documented, though opportunities for improving the quality and consistency of care remain (Wang *et al.*, 2016; Assefa *et al.*, 2019).

Kenya

In Kenya, the community health strategy was established in 2006 and revised in 2014 (Republic of Kenya Ministry of Health, 2014). The current policy includes two tiers of community health workers: community health volunteers (CHVs) and Community Health Extension Workers (CHEWs), the latter of which was only officially included in the national scheme of service in 2013 (Republic of Kenya, 2013). Though are both officially recognized, only the CHEWs are salaried government employees. Recommended ratios are one community health unit per approximately

5000 population, to be staffed by 5 CHEWs and 10 CHVs. Main responsibilities of both cadres are preventive and promotive health, with a focus on maternal and child health outcomes. CHEWs are linked to a primary health care facility (Level 2 or 3) and will supervise the CHVs; supervision is also provided directly by the sub-county health management team. Given devolution of health policy and decision making to the county (sub-national) level in the new Kenyan constitution of 2010, county policies on pay for the two cadres and key responsibilities vary (McCollum *et al.*, 2016, 2018). There have been many pilots of utilising CHVs to deliver various curative services but few of these have made it to policy and practice (Christoffersen-Deb *et al.*, 2015; Mushamiri *et al.*, 2015; Otiso *et al.*, 2017; Onono *et al.*, 2018; Gimaiyo *et al.*, 2019).

<u>Malawi</u>

The Malawian Ministry of Health has recently issued a new community health policy, the subject of much fanfare in the global UHC community (Malawi, 2017). This policy focuses on the Health Surveillance Assistants (HSAs) and improving role clarity and support for them; next, the country is focusing on is using this evidence to mobilize resources for expanding coverage and ensuring salaries and commodities under the new plan (Davey *et al.*, 2016; Barger *et al.*, 2017; Greco *et al.*, 2017). HSAs are nominated by their community, serving a population of approximately 1000, and receive 12 weeks of training focused on preventive health, family health and environmental health/sanitation. The HSAs also supervise CHVs and are supervised by senior HSAs, as well as by Environmental Health Officers and Community Nurses based at their link Health Facility (Kok *et al.*, 2016, 2018). Reporting is done on Form 1A and summarized in Form 1B before being entered into the DHIS2 at the district level. Several papers have been published examining the poor data quality in this community health system and possible means to improve that (Admon *et al.*, 2013; Joos and Silva, 2016; Yourkavitch *et al.*, 2016).

Mozambique

In Mozambique, the community health programme is implemented by Agentes Polivalentes Elementares (APEs). Although founded in the 1970s, the programme had nearly died out and was recently revitalized in 2010-11 to create a salaried cadre of community health workers (MISAU, 2010, 2011). APEs can be male or female and are elected by their communities and receive four months of residential training; this has been observed to be a barrier to equitable participation by both genders and men represent the majority of APEs (Steege *et al.*, 2018). Most of the care APEs provide should be preventive and promotive, though when commodities are available they also do integrated community case management of fever or iCCM for children under five years old; referral is another important function they provide (Davlantes *et al.*, 2019; Give *et al.*, 2019). APEs are supervised by link facility staff and district health management teams, but this is intermittent (Ndima *et al.*, 2015).

References

Admon, A. J. *et al.* (2013) 'Assessing and improving data quality from community health workers: a successful intervention in Neno, Malawi.', *Public health action*, 3(1), pp. 56–59. doi: 10.5588/pha.12.0071.

Assefa, Y. *et al.* (2019) 'Community health extension program of Ethiopia, 2003-2018: Successes and challenges toward universal coverage for primary healthcare services', *Globalization and Health*. BioMed Central Ltd., 15(1). doi: 10.1186/s12992-019-0470-1.

Barger, D. *et al.* (2017) 'Multi-country analysis of the cost of community health workers kits and commodities for community-based maternal and newborn care', *Health Policy and Planning*, 32(November), pp. i84–i92. doi: 10.1093/heapol/czx038.

Christoffersen-Deb, A. *et al.* (2015) 'Chamas for Change: an integrated community-based strategy of peer support in pregnancy and infancy in Kenya', *The Lancet Global Health*, 3, p. S22. doi: 10.1016/S2214-109X(15)70141-5.

Datiko, D. G. *et al.* (2019) 'Community participation and maternal health service utilization: lessons from the health extension programme in rural southern Ethiopia', *Journal of Global Health Reports*, 3, pp. 1–12. doi: 10.29392/joghr.3.e2019027.

Davey, S. *et al.* (2016) *Modelling the Cost of Community Health Services in Malawi : the Results of Piloting a New Planning and Costing Tool*.

Davlantes, E. *et al.* (2019) 'Malaria case management commodity supply and use by community health workers in Mozambique, 2017', *Malaria Journal*. BioMed Central, 18(1), p. 47. doi: 10.1186/s12936-019-2682-5.

Gimaiyo, G. *et al.* (2019) 'Can child-focused sanitation and nutrition programming improve health practices and outcomes? Evidence from a randomised controlled trial in Kitui County, Kenya', *BMJ Global Health*. BMJ Specialist Journals, 4(1), p. e000973. doi: 10.1136/bmjgh-2018-000973.

Give, C. *et al.* (2019) 'Strengthening referral systems in community health programs: A qualitative study in two rural districts of Maputo Province, Mozambique', *BMC Health Services Research*. BioMed Central Ltd., 19(1). doi: 10.1186/s12913-019-4076-3.

Give, C. S. *et al.* (2015) 'Exploring competing experiences and expectations of the revitalized

community health worker programme in Mozambique: an equity analysis', *Human Resources for Health*. BioMed Central, 13(1), p. 54. doi: 10.1186/s12960-015-0044-0.

Greco, G. *et al.* (2017) 'Malawi three district evaluation : Community- based maternal and newborn care economic analysis', (November), pp. 64–74. doi: 10.1093/heapol/czw079.

Joos, O. H. and Silva, R. (2016) 'Evaluation of a mHealth Data Quality Intervention to Improve Documentation of Pregnancy Outcomes by Health Surveillance Assistants in Malawi : A Cluster Randomized Trial Evaluation of a mHealth Data Quality Intervention to Improve Documentation of Pregnan', (January). doi: 10.1371/journal.pone.0145238.

Kane, S. *et al.* (2016) 'Limits and opportunities to community health worker empowerment: A multi-country comparative study', *Social Science & Medicine*. Pergamon, 164, pp. 27–34. doi: 10.1016/J.SOCSCIMED.2016.07.019.

Kok, M. C. *et al.* (2015) 'A qualitative assessment of health extension workers' relationships with the community and health sector in Ethiopia: opportunities for enhancing maternal health performance.', *Human Resources for Health*, 13(1). doi: 10.1186/s12960-015-0077-4.

Kok, M. C. *et al.* (2016) 'Health surveillance assistants as intermediates between the community and health sector in Malawi: exploring how relationships influence performance', *BMC Health Services Research*. BioMed Central, 16(1), p. 164. doi: 10.1186/s12913-016-1402-x.

Kok, M. C. *et al.* (2018) 'Does supportive supervision enhance community health worker motivation? A mixed-methods study in four African countries', *Health Policy and Planning*, (September), pp. 988–998. doi: 10.1093/heapol/czy082.

Kumar, M. B. *et al.* (2014) *Access to healthcare through community health workers in East and Southern Africa*. New York, NY, USA.

Kumar, M. B. *et al.* (2019) 'Is quality affordable for community health systems? Costs of integrating quality improvement into close-To-community health programmes in five low-income and middle-income countries', *BMJ Global Health*. BMJ Specialist Journals, 4(4), p. e001390. doi: 10.1136/bmjgh-2019-001390.

Lewin, S. *et al.* (2010) 'Lay health workers in primary and community health care for maternal and child health and the management of infectious diseases (Review)', *Cochrane Database of Systematic Reviews*, (3). doi: 10.1002/14651858.CD004015.pub3.www.cochranelibrary.com.

Mahmud, I. *et al.* (2015) 'Exploring the context in which different close-to-community sexual and reproductive health service providers operate in Bangladesh: a qualitative study', *Human Resources for Health*. BioMed Central, 13(1), p. 51. doi: 10.1186/s12960-015-0045-z.

Malawi, G. of the R. of (2017) *National Community Health Strategy 2017-2022*. Available at: http://www.chwcentral.org/sites/default/files/National_Community_Health_Strategy_2017-2022%2BFINAL.pdf.

McCollum, R. *et al.* (2016) 'Exploring perceptions of community health policy in Kenya and identifying implications for policy change', *Health Policy and Planning*. Oxford University Press, 31(1), pp. 10–20. doi: 10.1093/heapol/czv007.

McCollum, R. *et al.* (2018) 'Health system governance following devolution: comparing experiences of decentralisation in Kenya and Indonesia', *BMJ Global Health*. BMJ Specialist Journals, 3(5), p. e000939. doi: 10.1136/bmjgh-2018-000939.

Ministry of Health, F. (2007) *Health Extension Programme in Ethiopia*. Addis Ababa, Ethiopia.

Mireku, M. *et al.* (2014) *Context analysis: Close-to-community health service providers in Kenya*.

MISAU (2010) 'Programa de Revitalização dos Agentes Polivalentes Elementares', p. 37.

MISAU (2011) 'Guiao operacional para o programa dos agentes polivalentes elementares'.

Mushamiri, I. *et al.* (2015) 'Evaluation of the impact of a mobile health system on adherence to antenatal and postnatal care and prevention of mother-to-child transmission of HIV programs in Kenya', *BMC Public Health 2015 15:1*. BioMed Central, 15(1), p. 102. doi: 10.1186/s12889-015-1358-5.

Ndima, S. D. *et al.* (2015) 'Supervision of community health workers in Mozambique: a qualitative study of factors influencing motivation and programme implementation', *Human Resources for Health*. BioMed Central, 13(1), p. 63. doi: 10.1186/s12960-015-0063-x.

Nyirenda, L. *et al.* (2014) 'Report on the context analysis of close-to-community providers in Malawi.', *REACH Trust*.

Olaniran, A. *et al.* (2017) 'Who is a community health worker? – a systematic review of definitions', *https://doi.org/10.1080/16549716.2017.1272223*. Taylor & Francis. doi: 10.1080/16549716.2017.1272223.

Onono, M. *et al.* (2018) 'Using the RE-AIM framework to evaluate the implementation of

integrated community case management in Kenya', *Acta Paediatrica*, 107(Suppl. 471), pp. 53–62. doi: 10.1111/apa.14662.

Ormel, H. *et al.* (2019) 'Salaried and voluntary community health workers: exploring how incentives and expectation gaps influence motivation', *Human Resources for Health*. BioMed Central, 17(1), p. 59. doi: 10.1186/s12960-019-0387-z.

Otiso, L. *et al.* (2017) 'Decentralising and integrating HIV services in community-based health systems: a qualitative study of perceptions at macro, meso and micro levels of the health system', *BMJ Global Health*. BMJ Specialist Journals, 2(1), p. e000107. doi: 10.1136/bmjgh-2016-000107.

Republic of Kenya, D. of P. S. M. (2013) *Scheme of Service for Community Health Services Personnel*.

Republic of Kenya Ministry of Health (2014) 'Strategy for Community Health', pp. 1–44.

Steege, R. *et al.* (2018) 'How do gender relations affect the working lives of close to community health service providers? Empirical research, a review and conceptual framework', *Social Science & Medicine*. Pergamon. doi: 10.1016/J.SOCSCIMED.2018.05.002.

Tumbelaka, P. *et al.* (2018) 'Analysis of Indonesia's community health volunteers (kader) as maternal health promoters in the community integrated health service (Posyandu) following health promotion training', *International Journal Of Community Medicine And Public Health*, 5(3). Available at: http://ijcmph.com/index.php/ijcmph/article/view/2601/1830 (Accessed: 12 February 2018).

Wang, H. *et al.* (2016) *Ethiopia Health Extension Program: an institutionalized community approach for universal health coverage*. Washington, D.C.: The World Bank. doi: 10.1596/978-1-4648-0815-9.

Yourkavitch, J. *et al.* (2016) 'How do we know? An assessment of integrated community case management data quality in four districts of Malawi', *Health Policy Plan*, pp. 1–10. doi: 10.1093/heapol/czw047.
