# Supplementary material for: How do decision-makers use evidence in community health policy and financing decisions? A qualitative study and conceptual framework in four African countries
Source: Health Policy Plan. 2020 Jun 9;35(7):799–809. doi: 10.1093/heapol/czaa027 (PMC7487332; doi:10.1093/heapol/czaa027)
Supplement: czaa027_supplementary_data [file czaa027_supplementary_data.zip › MKumar_suppl material_2_discussion guide_clean.docx]

Selected questions from the discussion guide relevant to findings in this paper

Section A: General close-to-community experience

1. Describe broadly your experience with community health workers and programs. How long have you been working in this area? What makes you enjoy it? What are the biggest challenges?
2. What is the role of your organization in working with community health programs?
3. What is the quality of the community health services currently provided in the (geographic) areas where you work?

Section B: Financing CHWs

*[NB: this may be public/national, public/sub-national, NGO, external funds; please ensure probing for domestic allocation e.g. between curative vs. preventive care, between different geography in the country, embedding of external programs into routine practice as well as applications to external donors]*

1. Would you describe specifically any funding you give or generate related to community health.
   1. What is the evidence or information that underpins this decision? (priority setting)
2. Who applies for the funding and what is the application process like? How long does it take?
3. What is the decision-making process for funding CHW programs?
   1. Who decides and what criteria do they use?
   2. What is the evidence or information that underpins this decision?
   3. How do you decide where to give money (geographically)? Is this decided in advanced or based on applications?
4. Once a funding decision is made, how does the money get transferred? (Specifically: through domestic channels or through parallel programs/implementers?) Is this the same at all sites?

Section C: Case example of QI for CHWs

*[NB: take QI for community health as a case example of a program or intervention that might be uptaken into routine practice and explore evidence needs, use, and possible financing mechanisms]*

1. We touched briefly on quality of community health programs earlier. How do you understand the term ‘quality improvement’ in the context of community health?
   1. (*interviewers: ask for other groups make up the CH ecosystem):* How do you think communities see this? Supervisors/health systems? CHWs themselves?
2. Please describe any community health QI training and activities that you are an active participant in. If none, list any of which you are aware.

For each:

- 1. Please share how it is financed?
     1. If you are funding it, what made you fund QI?
     2. Is there a cost share between partners on this work? Who are they?
     3. What challenges were faced around costs?
     4. *(If donor financed)* who led the proposal development?
  2. How do you evaluate the success of your QI program?
     1. Did you conduct any economic evaluation and if so could you share that information?
  3. What do you think could build on this in the future?

1. What are the benefits/outcomes/impacts you expect(ed) from any/this QI work with CHWs?
   1. On the individuals involved?
   2. On the institutions or facilities participating?
   3. On the system more broadly?
   4. Do you believe these changes will be sustained over the next 5 years? 10 years? Why or why not?
2. What evidence would you like to see that these benefits are being realized?
   1. Are there any examples you could share about how that evidence has been effectively presented to you or by you?

*Probe for documents/reports/evaluations and ask if they can be shared*

1. What kind of change would be required to merit an (additional) investment of funds available to you in this area?
   1. What evidence would help you know that it was worth the investment?
   2. What degree of cost would be acceptable given that degree of change? – does QI deliver ‘bang for your buck’?
   3. What do you view as competing with this type of intervention for financing?
2. What do you think are the cost implications of QI for CH?
3. Are you/Do you think national policymakers are interested in funding QI? Why or why not?
4. Other than financing, what would be required to achieve sustained change in this area?
