# Supplementary material for: How do decision-makers use evidence in community health policy and financing decisions? A qualitative study and conceptual framework in four African countries
Source: Health Policy Plan. 2020 Jun 9;35(7):799–809. doi: 10.1093/heapol/czaa027 (PMC7487332; doi:10.1093/heapol/czaa027)
Supplement: czaa027_supplementary_data [file czaa027_supplementary_data.zip › MKumar_suppl material_3_codeframe.pdf]

Name	Sources	References	Created On	Created By	Modified On	Modified By	Color
coordination	20	48	19 Jan 2019 02:59	MBK	18 Mar 2019 11:09	MBK	
economic evaluation evi…	9	18	19 Nov 2018 05:23	MBK	18 Mar 2019 11:16	MBK	
evidence vs politics	12	19	10 Jan 2019 02:04	MBK	18 Mar 2019 13:00	MBK	
importance of EE at di…	5	6	10 Jan 2019 02:05	MBK	18 Mar 2019 11:46	MBK	
role of EE in policy fu…	7	9	10 Jan 2019 02:04	MBK	18 Mar 2019 12:58	MBK	
embedding	23	67	10 Jan 2019 02:06	MBK	3 Mar 2019 23:00	MBK	
CHS as part of health…	23	44	17 Jan 2019 05:53	MBK	18 Mar 2019 13:07	MBK	
discussion from quant…	6	7	10 Jan 2019 02:06	MBK	25 Jan 2019 09:09	MBK	
key stakeholders	10	13	10 Jan 2019 02:06	MBK	3 Mar 2019 22:59	MBK	
non-financial	23	33	10 Jan 2019 02:06	MBK	18 Mar 2019 13:13	MBK	
Equity	10	14	22 Jan 2019 02:42	MBK	18 Mar 2019 13:10	MBK	
Financing of CHS	18	48	19 Nov 2018 05:16	MBK	18 Mar 2019 13:00	MBK	
budget impact	1	2	18 Mar 2019 13:00	MBK	18 Mar 2019 13:01	MBK	
competition with QI fo…	19	27	10 Jan 2019 02:05	MBK	18 Mar 2019 13:04	MBK	
Cost-effectiveness	3	4	19 Nov 2018 05:19	MBK	18 Mar 2019 13:00	MBK	
Decisionmaker	12	22	19 Nov 2018 05:19	MBK	18 Mar 2019 11:16	MBK	
devolution	16	45	16 Jan 2019 23:41	MBK	18 Mar 2019 11:16	MBK	
Disinvestment	5	5	19 Nov 2018 05:19	MBK	18 Mar 2019 11:14	MBK	
Evidence for financing…	13	25	19 Nov 2018 05:19	MBK	18 Mar 2019 13:01	MBK	
external financing	15	40	25 Jan 2019 08:35	MBK	18 Mar 2019 12:59	MBK	
roles in financing of di…	24	51	14 Jan 2019 13:51	MBK	18 Mar 2019 12:59	MBK	
governance	1	1	3 Mar 2019 22:58	MBK	3 Mar 2019 22:58	MBK	
leadership	13	35	29 Jan 2019 08:04	MBK	18 Mar 2019 13:13	MBK	
parallel to health system	2	4	3 Mar 2019 22:52	MBK	18 Mar 2019 11:00	MBK	
QI for CHS	20	31	19 Nov 2018 05:16	MBK	18 Mar 2019 13:11	MBK	
Cases or examples of…	22	42	19 Nov 2018 05:16	MBK	18 Mar 2019 13:10	MBK	
community-facility lin…	18	32	19 Jan 2019 03:04	MBK	18 Mar 2019 13:07	MBK	
dissemination and lea…	3	4	18 Mar 2019 11:34	MBK	18 Mar 2019 13:14	MBK	
Evaluation of QI for CHS	7	10	19 Nov 2018 05:23	MBK	18 Mar 2019 13:02	MBK	
Perceived cost of QI f…	16	29	19 Nov 2018 05:18	MBK	18 Mar 2019 13:13	MBK	
Quality of CHS	24	58	19 Nov 2018 05:16	MBK	18 Mar 2019 12:55	MBK	
Accountability for qua…	12	13	19 Nov 2018 05:18	MBK	18 Mar 2019 11:05	MBK	
Measuring quality	26	55	19 Nov 2018 05:18	MBK	18 Mar 2019 13:08	MBK	

MoH structures for qu…	17	39	19 Jan 2019 02:56	MBK	18 Mar 2019 13:03	MBK
Patient experience or…	12	16	19 Nov 2018 05:18	MBK	18 Mar 2019 12:56	MBK
Policy for CHS	12	33	21 Jan 2019 12:48	MBK	18 Mar 2019 13:05	MBK
Supervision	22	47	14 Jan 2019 13:48	MBK	18 Mar 2019 13:13	MBK
task-shifting	13	16	19 Nov 2018 05:22	MBK	18 Mar 2019 10:52	MBK
targets and performance…	2	5	18 Mar 2019 11:41	MBK	18 Mar 2019 12:56	MBK
UHC agenda	22	44	10 Jan 2019 02:05	MBK	18 Mar 2019 13:10	MBK
Value of QI	10	15	10 Jan 2019 02:02	MBK	18 Mar 2019 13:14	MBK
Benefits of QI for CHS	29	119	19 Nov 2018 05:17	MBK	18 Mar 2019 13:13	MBK
Accountability benefit	3	3	14 Jan 2019 14:00	MBK	18 Feb 2019 04:58	MBK
Adherence benfit	3	3	14 Jan 2019 14:01	MBK	18 Mar 2019 11:31	MBK
Allocation efficienc…	6	6	14 Jan 2019 14:00	MBK	22 Jan 2019 02:48	MBK
Benefits they would…	2	3	10 Jan 2019 02:02	MBK	20 Jan 2019 13:19	MBK
client satisfaction	2	2	18 Mar 2019 11:17	MBK	18 Mar 2019 13:02	MBK
Competition benefit	2	2	14 Jan 2019 14:03	MBK	18 Feb 2019 04:59	MBK
Data quality benefit	12	21	19 Jan 2019 04:03	MBK	18 Feb 2019 13:08	MBK
Evidence of benefits	9	10	19 Nov 2018 05:17	MBK	18 Feb 2019 13:05	MBK
Health benefit	13	17	19 Jan 2019 02:54	MBK	18 Mar 2019 13:02	MBK
How can we measu…	0	0	10 Jan 2019 02:03	MBK	10 Jan 2019 02:03	MBK
Ownership benefit	10	11	14 Jan 2019 14:01	MBK	3 Mar 2019 23:00	MBK
What evidence would…	7	8	10 Jan 2019 02:03	MBK	26 Feb 2019 03:22	MBK
